# The Application of Mesenchymal Stem Cells in the Treatment of Liver Diseases: Mechanism, Efficacy, and Safety Issues

**DOI:** 10.3389/fmed.2021.655268

**Published:** 2021-05-31

**Authors:** Ya Yang, Yalei Zhao, Lingjian Zhang, Fen Zhang, Lanjuan Li

**Affiliations:** State Key Laboratory for Diagnosis and Treatment of Infectious Diseases, National Clinical Research Center for Infectious Diseases, Collaborative Innovation Center for Diagnosis and Treatment of Infectious Diseases, The First Affiliated Hospital, College of Medicine, Zhejiang University, Hangzhou, China

**Keywords:** mesenchymal stem cells, liver diseases, mechanism, efficacy, safety

## Abstract

Mesenchymal stem cell (MSC) transplantation is a novel treatment for liver diseases due to the roles of MSCs in regeneration, fibrosis inhibition and immune regulation. However, the mechanisms are still not completely understood. Despite the significant efficacy of MSC therapy in animal models and preliminary clinical trials, issues remain. The efficacy and safety of MSC-based therapy in the treatment of liver diseases remains a challenging issue that requires more investigation. This article reviews recent studies on the mechanisms of MSCs in liver diseases and the associated challenges and suggests potential future applications.

## Introduction

After Friedenstein et al. (1) first isolated and identified stromal cells from bone marrow in 1966, mesenchymal stem cells (MSCs) were isolated from various tissues, such as the umbilical cord (2, 3), placenta (4), adipose tissue (5, 6), amniotic fluid (7, 8), menstrual blood (9, 10), and dental pulp (11). The immunomodulatory properties, limited self-renewal capacity, and multi-lineage development of MSCs make these cells ideal candidates for clinical applications in different diseases (12). The low inherent immunogenicity of MSCs guarantees transplant safety (13). Even HLA-mismatched MSCs could be used for many clinical applications, especially for stem cell-based therapies (14). Moreover, the ability of these cells to home to specific organs and lesions is the key to the curative effect of MSC transplantation (13). MSC surface chemokine receptors, such as CCR1, CCR4, CCR7, CXCR5, and CCR10, are involved in the migration of MSCs into injured tissues along chemokine gradients (15). MSCs are administered to patients by various routes, such as intravascular injection and local transplantation, to alleviate diseases (16–18).

Liver diseases are a global health issue that cause a large number of deaths every year (19, 20). Liver transplantation, which is recommended as the only effective treatment method available for advanced liver diseases, is limited by high costs and a shortage of donor livers (21). MSC transplantation brings new hope to the treatment of liver diseases. Despite their different etiologies, symptoms and physiological processes, multiple kinds of life-threating liver diseases can be effectively treated by cell-based therapy, as three decades of clinical and preclinical studies proved. Among the most used source of cells for allogenic/autologous transplantation, the two most largely clinically infused cells are undoubtedly primary hepatocyte and MSCs. MSCs have been administered in hundreds of clinical trials during the past decades, and for a plethora of indications (Table 1). Although many laboratory and clinical trials have confirmed the efficacy of MSCs in a variety of diseases, there are still no guidelines to regulate MSC clinical applications (29–31).

**Table 1:** Clinical trials of MSCs in liver diseases.

**Liver disease**	**MSC source**	**Follow-up**	**Clinical results**	**Possible related mechanisms**	**References**
Chronic hepatitis B cirrhosis	UC-MSC	48 weeks	Improve liver function and reduce ascites	Regulate HSC activation; reduce resistance to portal flow	(22)
Acute liver allograft rejection	UC-MSC	12 weeks	Decrease ALT levels; improve allograft histology	Increase Treg/Th17 ratio and downregulate CD41 T-cell	(23)
Hepatitis B virus-related Acute-on-chronic liver failure	BM-MSC	24 weeks	Increases survival rate; improve liver function; decrease severe infections incidence	Immunomodulation and anti-inflammation effect	(24)
HCV positive end-stage liver disease	BM-MSC	6 months	Improve liver functions and ascites; improve Child-Pugh and performance score	Downregulate collagen matrix formation; increase serum S-albumin level	(25)
Alcoholic cirrhosis	BM-MSC	12 months	Improved histologic fibrosis and liver function; improve ascites and encephalopathy	Mechanism for fibrosis reduction is not elucidated	(26)
Decompensated cirrhosis	BM-MSC	12 months	Improvement in MELD score and liver function; no obvious effect in improving histologic fibrosis	MSCs transplantation is probably not effective in decompensated cirrhosis	(27)
Ischemic-type biliary lesion	UC-MSC	2 years	Improve liver performance; reduce need for interventional therapies	The related mechanism is not elucidated	(28)

The purpose of this review is to summarize and critically discuss the therapeutic effects and related mechanisms of MSCs derived from different sources in the treatment of liver diseases, as well as indicate present issues including efficacy, safety and available routes of administration in clinical MSC therapy trials. We hope this review will provide a reference for future clinical trials and applications.

## Role of MSC in Tissue Repair and Regeneration

MSCs can be induced to differentiate into a variety of cell lineages, including adipocytes, osteoblasts, chondroblasts, and hepatocyte-like cells (HLCs), *in vitro* (32). This characteristic initially gave hope to regenerative medicine. Previous studies have shown that MSCs derived from different tissues, such as bone marrow (BM-MSCs) (33), adipose tissue (AT-MSCs) (34), menstrual blood (MenSCs) (35), and amniotic fluid (AF-MSCs) (34), can differentiate into HLCs *in vitro*. Clinical and laboratory trials suggested that MSCs could significantly improve liver cell regeneration in different kinds of liver diseases. Despeyroux et al. (36) showed that AF-MSCs improved liver regeneration and survival after 80% hepatectomy in mice. However, by tracking green fluorescent protein-expressing MenSCs, Chen et al. (37) showed that transplanted cells were recruited to injured liver sites in carbon tetrachloride (CCl_4_)-induced liver fibrosis mouse models, but few cells differentiated into HLCs. According to Shi et al. (38) human-derived hepatocytes constituted only 4.5% of total pig hepatocytes after intraportal vein infusion of BM-MSCs (3 × 106 cells/kg) in D-galactosamine (D-gal)-induced model pigs. von Bahr et al. (39) demonstrated that even minimally expanded BM-MSCs showed limited long-term engraftment and no ectopic tissue formation upon intravascular infusion. This means that MSCs may mainly promote liver regeneration through mechanisms other than differentiation into HLCs.

Several groups have showed that bone marrow transplants in mice led to the generation of liver cells bearing the donor marker, and demonstrated that this event might not due to transdifferentiation of MSCs into hepatic lineage cells. Moreover, this suggest is challenged by the scientists who were unable to reproduce the transdifferentiation of not only MSCs, but also hematological cells into non-hematological ones. Willenbring et al. (40) found that the transplantation into Fah(–/–) mice of lineage-committed granulocyte-macrophage progenitors or bone marrow-derived macrophages resulted in the robust production of bone marrow-derived hepatocytes by cell fusion, which provides potential for organ regeneration. Wang et al. (41) concluded that bone marrow-derived hepatocytes arised from cell fusion and rather than differentiation of hematopoietic stem cells. According to Camargo et al. (42), hematopoietic myelomonocytic cells are the major source of hepatocyte fusion partners. Such melting event is not occurring only to bone marrow derived cells. Other types of cells, such as perinatal MSCs, have also been reported to fuse with hepatocytes (43). Okamura et al. (44) found that developing monkey embryonic stem cells could repopulate injured mouse liver by fusing with recipient mouse hepatocytes.

Studies have demonstrated that MSCs can stimulate liver cell proliferation and inhibit hepatocyte apoptosis. In a trial that used D-gal-induced rat models of acute liver injury, MSC therapy resulted in a 90% reduction in apoptotic hepatocellular death and a three-fold increase in the number of proliferating hepatocytes (45). Efimenko et al. (46) showed that MSC-conditioned medium (MSC-CM) attenuated CCl_4_-induced early apoptosis in C57/BL6 mouse hepatocytes through activation of FGL1. According to Chen et al., MSC-conditioned medium injection could prevent radiation-induced liver injury by protecting sinusoidal endothelial cells (47). These results indicated that MSCs protect liver injury and stimulated hepatocyte proliferation by paracrine effects.

Different liver diseases display unique pathophysiological manifestations according to their etiology (48). MSCs may promote liver regeneration through different mechanisms in different disease models.

Accumulating evidence supports that MSCs play therapeutic roles in a paracrine manner, especially through trophic factors (49–52). Hepatocyte growth factor (HGF) and vascular endothelial growth factor (VEGF) are the most widely reported trophic factors secreted by MSCs (46). Antibody array results showed that MSC-derived exosomes (MSC-EXs) contain measurable HGF (53). HGF is widely known to be a crucial factor in the positive regulation of hepatocyte proliferation (54). VEGF secreted by MSCs contributes to the recovery of liver damage (55). Recent studies have also demonstrated the role of miRNAs in MSC-induced liver regeneration. According to Kim et al. (56), rno-miR-122-5p is closely related to the therapeutic efficacy of placenta-derived mesenchymal stem cells (PD-MSCs) in liver tissues. PD-MSCs stimulate hepatocyte proliferation by activating the interleukin 6 (IL-6) signaling pathway through the regulation of rno-miR-21-5p. Hyun et al. (57) showed that microRNA125b-mediated regulation of Hh signaling contributed to liver regeneration that was promoted by chorionic plate-derived mesenchymal stem cells (CP-MSCs) in CCl_4_-induced rats.

## Mechanism of the Antifibrotic Effect of MSC

Liver fibrosis is characterized by the deposition of extracellular matrix (ECM), including collagen I, collagen III and collagen IV (58). The activation of hepatic stellate cells (HSCs) plays a crucial role in this process (55). Activated HSCs proliferate and transform into myofibroblasts (59, 60). Myofibroblasts synthesize ECM and release large amounts of TIMPs, which can reduce ECM degradation by inhibiting interstitial collagenase activity and ultimately induce ECM accumulation (61). Multiple signaling pathways, such as TGF-β/Smad, Ras/ERK, Notch, and Wnt/β-catenin, are involved in HSC activation (62–65). Kupffer cell activation is an important factor that induces HSC activation during chronic liver injury (66). Kupffer cells are resident macrophages in the liver. Activated Kupffer cells release large amounts of soluble mediators, such as oxidants, cytokines, and proteinases, which can affect HSC proliferation, migration, and differentiation (67).

Epithelial-to-mesenchymal transition (EMT) and mesenchymal-to-epithelial transition (MET) are important contributors to liver cirrhosis (68). Epithelial cells in the chronic injured liver undergo EMT, which makes these cells exhibit fibroblastic features and move into the hepatic mesenchyme. Then, these cells undergo MET to differentiate into hepatocytes or cholangiocytes to repair injured tissue. However, the microenvironment of the injured liver may upregulate the EMT, which stimulates fibrogenic repair and causes liver fibrosis (69).

MSCs have significant effects on liver fibrosis. *In vitro* and *in vivo* experiments demonstrated that MSCs mainly exert antifibrotic effects by paracrine mechanisms (70). Secretomes or the culture medium of MSCs could also significantly suppress liver fibrosis (71). MSC transplantation could alleviate liver fibrosis and reduce the expression of transforming growth factor-β1 (TGF-β1), Smad2, collagen type I, and α-SMA, and pathological examination showed reduced liver fibrosis areas (72). According to Jang et al. (73), BM-MSCs could reduce hepatic collagen distribution by suppressing the TGF-β/Smad signaling pathway in TAA-induced cirrhosis rat models. An et al. (74) found that the secretomes of UC-MSCs contained high levels of milk fat globule-EGF factor 8 (MFGE8). This factor could downregulate TGF-β1 receptor levels by binding to αβ integrin on hepatic stellate cells (HSCs), thereby strongly inhibiting the activation of primary human HSCs. MSCs can also exert antifibrotic effects through the Wnt/β-catenin pathway. According to Rong et al., BM-MSC-derived exosomes (BM-MSCs-Exs) could suppress HSC activation by inhibiting the expression of Wnt/β-catenin pathway components, including peroxisome proliferator activated receptor γ (PPARγ), Wnt3a, Wnt10b, β-catenin, WISP1, and Cyclin D1 (75).

Chai et al. (76) transfused UC-MSCs into dimethylnitrosamine (DMN)-induced liver fibrosis model rats and found that UC-MSCs alleviated liver fibrosis by increasing IL-4 levels and promoting the mobilization of Kupffer cells. UC-MSC-mediated regulation of Kupffer cells was demonstrated in an *in vitro* coculture system. According to Ohara et al. (77), amnion-derived mesenchymal stem cell-derived EVs (AMCS-EVs) could significantly inhibit HSC activation and decrease the number of Kupffer cells (KCs) in the livers of rats with liver fibrosis induced by CCl_4_.

On the other hand, MSCs can regulate the EMT-MET balance in fibrogenic liver tissue. According to Li et al., UC-MSC-Ex transplantation reduced the expression levels of collagen types I and III, TGF-β1 and phosphorylated Smad2 by inhibiting EMT activation in CCl_4_-induced liver fibrosis models (78). TGF-β has been demonstrated to activate fibrogenic EMT through the RAS and mitogen-activated protein kinase (MAPK) pathways (79, 80). To date, RAS-responsive element binding protein 1 (RREB1) has been identified as a key partner of TGF-β-activated SMAD transcription factors associated with the EMT in liver fibrosis (75). Considering the inhibitory effects of MSCs on TGF-β, MSCs may inhibit the EMT by regulating TGF-β activity, which requires further examination.

Besides, MSC applications are reported to improve liver fibrosis related complications, which are the mainly causes of death in clinic. According to Zhang et al. (22), UC-MSC transplantation could not only lead to the regression of liver fibrosis in patients, but also reduce related ascites. A meta-analysis showed that BM-MSC transplantation could improve ascites and encephalopathy in patients with chronic liver disease especially liver fibrosis (22). As to Pietrosi et al. (81), human amniotic membrane-derived mesenchymal stromal (hAMSCs) could improve hepatic microvascular dysfunction and portal hypertension, which are responsible for the complications defining clinical decompensation. Vitro experiment revealed that sinusoidal cell phenotype ameliorated when co-cultured with hAMSCs. However, the related mechanisms still need further explorations.

## Mechanism of the Immunomodulatory Effects of MSC

**Figure 1:**
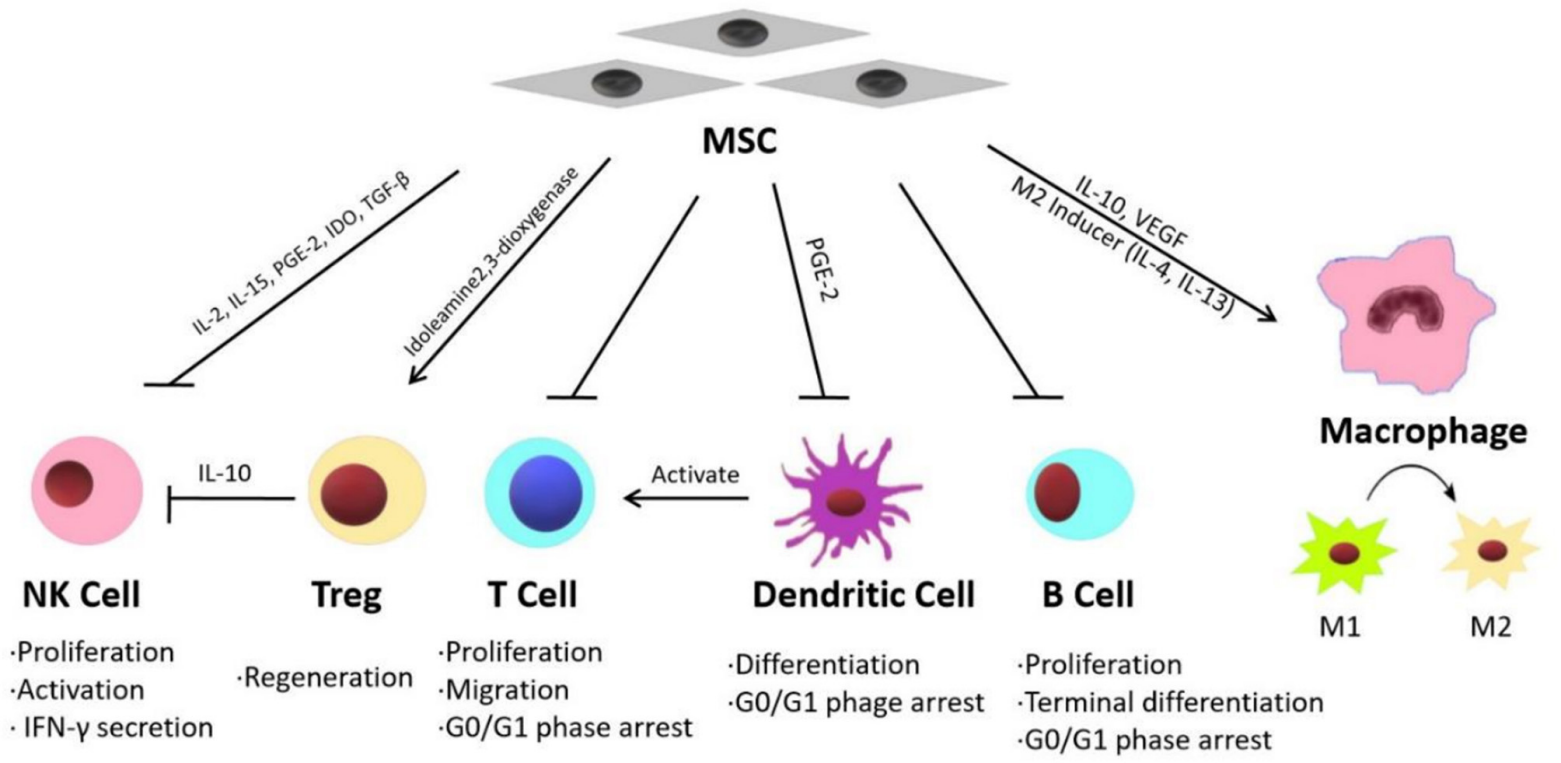
The immunomodulatory properties of MSC and related mechanisms.

Inflammatory reactions are widely detected in injured liver tissues and are considered the primary causes of fibrosis and hepatic function failure (82–84). Recent studies have shown that MSC therapy can reduce inflammation in liver diseases through different mechanisms. Some published studies have indicated that the effects of MSC treatment on various acute and chronic liver diseases are mainly mediated by their immunomodulatory properties (Figure 1) (15).

MSCs exhibit immunomodulatory functions through paracrine mechanisms (85). MSCs can release multiple immunosuppressive factors, such as IL-10, VEGF, and TGF-β (86–88). Winkler et al. identified the factors secreted by BM-MSCs and AT-MSCs through protein arrays and identified related pathways through biomathematical analyses. The results showed that many cytokines are involved in innate immunity and inflammatory pathways, including the JAK-STAT signaling pathway and Toll-like receptor pathway (89). On the other hand, MSCs can modulate the inflammatory microenvironment by suppressing the expression of inflammatory factors such as IFN, IL-1 and TNF-α (90). Furthermore, MSCs exhibit immunoregulatory activities by balancing the functions of different innate and adaptive immune cells, including natural killer cells (NK cells), T regulatory cells (Tregs), T lymphocytes, B lymphocytes, macrophages and dendritic cells (DCs).

According to DelaRosa et al., AT-MSCs and BM-MSCs inhibit IL-2- and IL-15-induced NK cell proliferation, as well as PGE-2- and IDO-induced NK cell activation. *In vitro* experiments showed that MSCs could modulate the secretion of IFN-γ by NK cells through the action of soluble factors such as indoleamine 2,3-dioxygenase (91).

Tregs play an important role in fulminant hepatitis. Tregs are necessary for the suppression of immune cell-mediated hepatocyte damage during fulminant hepatitis (92). MSCs significantly promote the regeneration of Tregs both *in vivo* and *in vitro*, thereby inhibiting immune cell activation (93). According to Qu et al., BM-MSC transplantation significantly attenuated immune-mediated liver injury and controlled virus levels in hepatitis B virus (HBV)-infected mice. Their study showed that BM-MSC-derived TGF-β suppressed the expression of natural killer group 2 member D (NKG2D), an important receptor required for NK cell activation in the liver in HBV-infected mice, thereby influencing innate immunity and limiting immune-mediated liver injury (94).

MSCs suppress T lymphocyte proliferation induced by mitogens, and CD3 and CD28 antibodies, as well as allogeneic antigens, both *in vitro* and *in vivo*. T lymphocytes can be arrested in the G0/G1 phase of the cell cycle by MSCs through the downregulation of cyclin D2 (95). Wang et al. (96) demonstrated that MSC treatment could reverse nonalcoholic fatty liver disease (NAFLD) by suppressing the activation of cluster of differentiation (CD) 4+ T lymphocytes in mouse spleens. MSCs have been shown to suppress B lymphocyte proliferation and terminal differentiation. According to Yi and Song MSCs inhibit B lymphocytes by arresting these cells in the G0/G1 phase of the cell cycle (92).

DCs play an important role in the initiation, maintenance and regulation of immune reactions by stimulating antigen-specific T cell activation (14). According to Ramasamy et al. (97), human BM-MSCs inhibit the differentiation of DCs by blocking the synthesis of cyclin D2 in monocytes, thereby arresting DCs in the G0/G1 phase of the cell cycle. Zhang et al. showed that MSCs effectively attenuated *Propionibacterium acnes* (*P. acnes*)-primed and lipopolysaccharide (LPS)-induced liver injury in mice and increased the survival rates of the animals by regulating DC differentiation. MSCs induced the differentiation of a distinct, functional CD11c1MHCIIhi CD80loCD86lo regulatory DC population from CD11c1B2202 DC precursors by secreting PGE2 through a PI3K-dependent pathway. This group of DCs suppressed Th1 cells while inducing Treg proliferation (98).

Two kinds of macrophages participate in the immune response during liver injury, local macrophages (Kupffer cells) and circulating macrophages. As a response to various inflammatory signals during the progression of liver injury, macrophages undergo classic activation (proinflammatory M1) and alternative activation (anti-inflammatory M2) (99). MSCs can regulate M1/M2 balance in macrophages. According to Cho et al., in bone marrow-derived macrophages co-cultured with MSCs, the M1 markers significantly reduced while the M2 markers increased. The result suggested that MSCs could promote the shift of the macrophage phenotype from M1 to M2 (100). Li et al. (101) suggested that MSCs exhibit therapeutic effect in liver sterile inflammatory injury by leading to reprograming macrophage polarization toward anti-inflammatory M2 phenotype through Hippo pathway. UC-MSC-Exs were shown to inhibit the expression of NLRP3, caspase-1, IL-1β, and IL-6 in LPS-stimulated RAW 264.7 macrophages (102).

Liver transplantation is considered the only effective treatment for end-stage liver diseases; however, subsequent rejection, especially acute graft-vs.-host disease (aGVHD), is the leading cause of surgical failure and postoperative death in patients (103). Various immune cells, including T cells, DCs, Tregs, and NK cells, mediate the occurrence and development of rejection (104). Accumulating evidence has demonstrated that MSC transplantation can significantly attenuate the severity of aGVHD due to the immunomodulatory effects of MSCs (104–106). However, the cytological and molecular mechanisms still require further exploration.

## Improved MSC Treatment Efficacy (Factors Influencing the Efficacy of MSC)

Although MSC treatment has shown significant effects in liver diseases, researchers are still exploring ways to increase the efficacy of MSC applications. According to previous studies, many factors can influence the efficacy of MSCs. Regardless of expansion protocol, MSCs undergo replicative senescence in culture, with obvious repercussion on therapeutical effects. Based on the study of telomere length, Baxter et al. (107) found that even protocols that involve minimal expansion induce a rapid aging of MSCs, which may influence MSC phenotype and paracrine potential. In AD-MSCs, the expression of the anti-inflammatory cytokine IL-10 showed substantial differences between P7 and P9, with a consistent decrease in mRNA expression (77). In BM-MSCs, compared with those of P1, the expression levels of IL-6 and VEGF were much higher in P5 (108). According to Choi et al., gradual decreases in IL-6 and VEGF expression levels during the long-term culture of MSCs may be related to reductions in the differentiation potential and proliferation of MSCs. The authors suggested that MSCs at earlier passages were more suitable for therapy due to their stability and more potent anti-inflammatory properties than cells at later passages (109). Another study stated that age reduced human MSC-mediated T cell suppression (110). Although the mechanisms of the impact of senescence on the immunomodulatory activity of MSCs are still not clear, multiple studies have demonstrated that senescence due to both donor age or multiple passages impacts the immunomodulatory properties of MSCs (111, 112). These results indicated that optimizing the criteria for the selection of MSC donors and low-passage MSCs could enhance the cell transplantation efficacy.

Pretreatment before application is the most common method to improve the therapeutic efficacy of MSCs *in vitro* and *in vivo*. Culture conditions significantly influence MSC phenotype. A hypoxic culture environment can contribute to the maintenance of MSC proliferation, differentiation and metabolic balance (113). According to Kojima et al., compared with those cultured in normal conditions, BM-MSCs cultured under hypoxic conditions showed greater therapeutic effects in mice with liver cirrhosis (114). Yu et al. found that hypoxia preconditioning enhanced the expression of VEGF in BM-MSCs *in vitro*. These pretreated MSCs exhibited improved regenerative effects in rat massive hepatectomy models (115).

Melatonin is an endogenous indoleamine produced and released into the blood circulation by the pineal gland (116). In addition to regulating biorhythms, melatonin can also play an antiaging role due to its antioxidant effects (117). Mohsin et al. (118) found that pretreating MSCs with injured liver tissue resulted in high expression of albumin, cytokeratin 8, 18, TAT and HNF1α, thereby improving the antifibrotic effect of MSCs in CCl_4_-induced mice. Fang et al. (119) showed that AD-MSCs pretreated with melatonin showed enhanced beneficial effects in canine acute liver injury. *In vitro* experiments showed that melatonin pretreatment improved the survival of AD-MSCs by activating Nrf2 through the MT1/MT2 receptor pathway, stimulating ERAD, inhibiting NF-κB and ERS, and alleviating AD-MSC senescence (120). Another study indicated that melatonin pretreatment enhanced the homing capacity of BM-MSCs in a rat model of liver fibrosis (121).

Recently, an increasing number of studies have focused on the role of polymer materials in promoting the efficacy of MSCs in liver diseases. Salem et al. (122) reported that pretreatment with growth factors in the presence of nanofibers promoted the homing, repopulation and hepatic differentiation abilities of MSCs, thereby increasing the efficacy of MSCs in liver fibrosis. As the most frequently used polymer combinations for cell microencapsulation, hybrid poly (ethylene glycol)-alginate hydrogels have been chosen to pretreat MSCs to protect cells against larger compounds such as circulating antibodies, as well as immune and inflammatory cells, without blocking nutrient and metabolite exchange (123). Studies have shown that hybrid poly (ethylene glycol)-alginate hydrogel microencapsulation significantly improved MSC efficacy in ALF and liver fibrosis (124, 125).

In addition to MSC pretreatment, gene modification is also widely used to improve the therapeutic effects of MSCs in liver diseases. Overexpression of c-Met in BM-MSCs could improve the homing of BM-MSCs to injured liver tissue, thereby promoting the efficacy of BM-MSC therapy for ALF repair in rats (126). Various studies have demonstrated that HGF-overexpressing MSCs present an increased ability to treat liver injuries by promoting liver regeneration (127–129). On the other hand, HGF-overexpressing MSCs have longer telomeres, as well as increased mtDNA replication, which leads to increased ATP generation (130).

**Figure 2:**
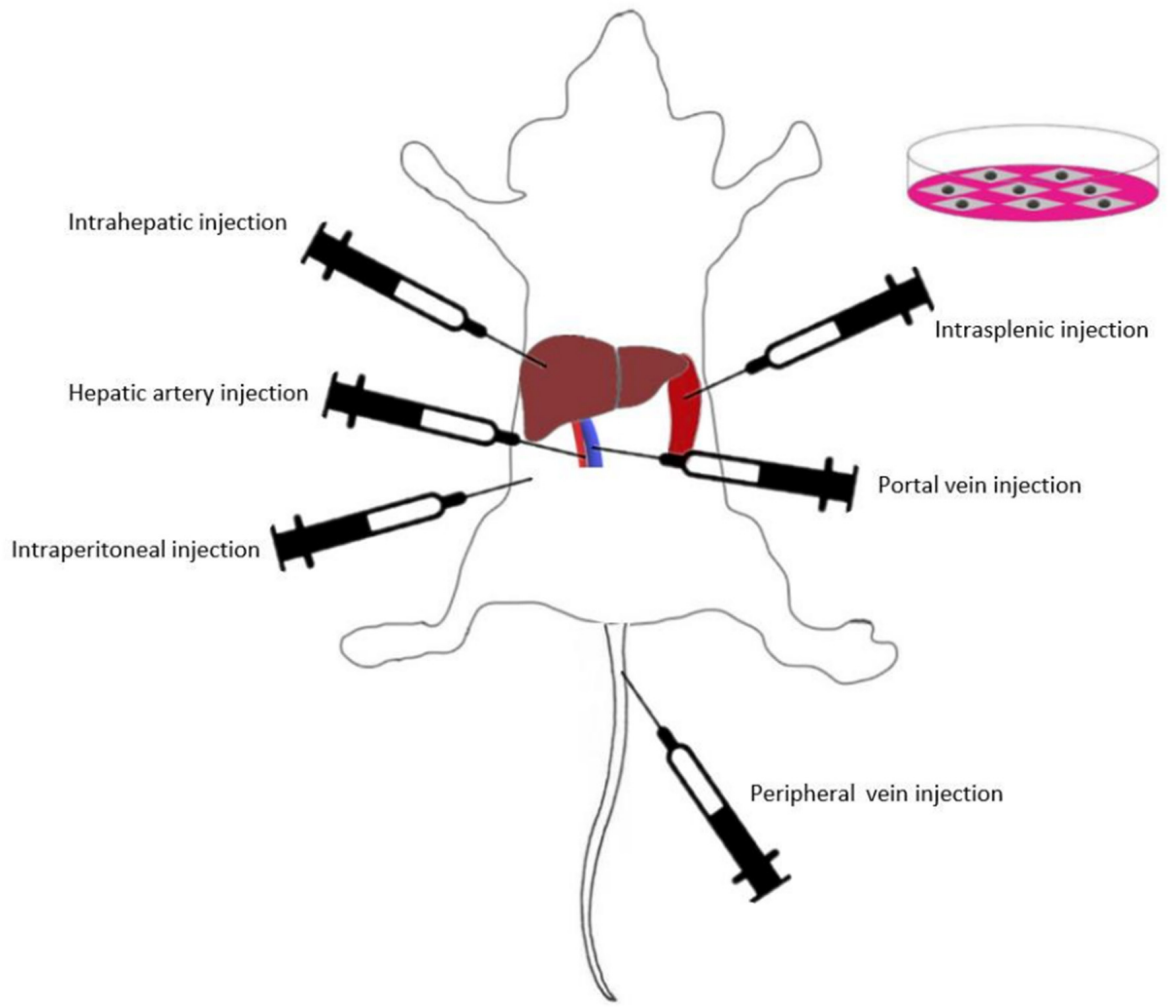
Various routes of MSC transplantation in animal models and clinical trials.

Presently, various routes of MSC transplantation have been proven to be curative in different liver injuries (Figure 2). Intravascular infusion is the most popular route for MSC transplantation in animal models and clinical trials (131). Intravascular infusion of MSCs typically proceeds via portal vein injection, hepatic artery injection or peripheral vein injection (132). In addition, local transplantation methods such as intraperitoneal injection, intrasplenic injection and intrahepatic injection are also widely used in MSC efficacy studies (16, 133–135).

To date, there have been few comparative studies on different methods of MSC transplantation. Sun et al. (132) assessed the efficacy of four BM-MSC transplantation methods (hepatic artery injection, portal vein injection, vena caudalis injection, and intraperitoneal injection) in a rat model of D-galactosamine (D-gal)/lipopolysaccharide (LPS)-induced ALF. The results showed that intravascular infusion was significantly more effective than intraperitoneal injection, while the selection of blood vessels as the implantation pathway did not affect the transplantation outcomes. Idriss et al. (16) compared the efficacy of intravenous and intrasplenic BM-MSC transplantation in CCl4-induced liver fibrosis model rats. The authors found that the intravenous route was more effective than the intrasplenic route in suppressing the gene expression levels of IL-1β, IL-6, and INF-γ. This result indicated that compared with the intrasplenic route, intravenous BM-MSC injection was an efficient and appropriate route for BM-MSC transplantation in liver fibrosis. Zhao et al. showed that compared with the intrahepatic and intraperitoneal injection groups, the BM-MSC intravenous injection group had the highest number of MSCs that migrated into liver lobules in CCl4-induced fibrosis model rats. IL-10 levels were highest in the intravenous group, whereas IL-1β, IL-6, TNF-α, and TGF-β were significantly lower than those in the other groups (136). These studies indicated that intravascular infusion was very suitable for BM-MSC transplantation. According to a study of UC-MSCs, UC-MSC transplantation via the tail vein had similar therapeutic efficacy compared with that of intrahepatic injection (137). The present study on MSC transplantation routes has some potential limitations. Further explorations are needed to determine the optimal application routes of MSCs from different sources, and the related mechanisms are still not completely understood.

Although various methods have been demonstrated to successfully improve MSC efficacy in liver diseases in animal models, current culture conditions (both in term of medium composition and supplements, adhesion to ECM selective proteins, and exposure to inflammatory signals) significantly influence MSC phenotype and paracrine potential. That's why further explorations are still needed for clinical applications in the future.

## Safety Issues Associated With MSC Transplantation

Large numbers of *in vitro* and *in vivo* trials have demonstrated the capacity of MSCs to promote regeneration, antifibrosis, and immunomodulation, as well as the significant effects of MSCs in various liver injury models. These findings opened up possibilities for the clinical application of MSCs in liver diseases. A growing number of clinical trials have demonstrated the therapeutic effect of MSCs in patients with liver diseases, especially ALF, cirrhosis, and GVHD (23, 138, 139). However, safety is still the most concerning issue in MSC clinical applications. Despite rash and fever (37–38°C) in several cases that resolved without additional treatment (24), no significant adverse effects were reported in most clinical trials. A series of meta-analysis results also proved the therapeutic efficacy and safety of MSCs from different sources in patients with ALF, liver cirrhosis and end-stage liver disease associated with HBV and HCV (22, 140–142).

MSCs have been shown to have the ability to migrate and integrate into tumor tissue (143), but the effect of MSCs on hepatocellular carcinoma cells *in vitro* and *in vivo* is still controversial. According to Zhao et al. (144), AD-MSC-CM inhibited proliferation and promoted cell death in a hepatocellular carcinoma cell line *in vitro*. Some studies have indicated that BM-MSCs can promote the migration and invasion of hepatocellular carcinoma cells (145, 146). Moreover, investigators have documented the influence of MSC culture on genetic instability and tumorigenicity (147). Rosland et al. (148) found that malignant transformation occurred in 45.8% of the human MSCs after long-term cultures (5–106 weeks). According to Ren et al. (149), MSCs derived from adult cynomolgus monkeys could transform spontaneously into highly tumorigenic transformed mesenchymal cells (TMCs) after cultured *in vitro*. Although many researchers announced that MSC transplantation was not likely to cause tumors after following up with patients for up to 11 years and 5 months (150), it is still not clear how MSCs influence tumorigenesis and development in patients.

Some researchers also indicated the risk of thrombosis and embolization that occurred during intravascular MSC administration due to the incompatibility of MSCs with the innate immune cascade systems of the blood. During a clinical trial of 11 patients with liver-based metabolic disorders, one patient exhibited a thrombogenic event after MSC infusion, and four patients were observed to have significant decreases in platelet and increases in D-dimer levels at the end of MSC infusion, which spontaneously normalized after 7 days. So Coppin et al. recommend anticoagulants combined with MSC infusion to limit infusion-related thrombogenesis to subclinical levels in patients (151). Moll et al. suggested that all cellular therapies should be subjected to hemocompatibility screening before intravascular infusion to ensure patient safety (152).

These studies emphasize that the regulation of MSC clinical applications still needs further exploration, evaluation and optimization. Moll et al. (131) indicated that comprehensive safety evaluations were essential before use in humans, and new clinical guidelines were needed to standardize MSC clinical treatment strategies.

## Conclusions

A large number of studies have demonstrated that MSCs exert therapeutic effects in liver diseases by promoting regeneration, regulating immunity, and inhibiting fibrosis. Further studies are ongoing to determine the related mechanisms and explore strategies to enhance MSC efficacy. In addition to pretreatment and gene modification of MSCs, the extraction and applications of MSC-CM and MSC-EV are also under intense study.

Although the application of MSCs from various tissue sources have entered clinical trials, several concerns remain, such as the low MSC survival rate, as well as the risk of carcinogenesis, thrombosis, and embolization. Furthermore, strict standards are needed to regulate MSC source selection, culture medium composition, culture conditions, delivery routes, doses, course of treatment, indications of application, and so on. In this review, we suggest that formulating and following treatment guidelines is the most effective way to avoid treatment risks and improve treatment efficacy.

## Author Contributions

LL, YY, and YZ designed and wrote the manuscript. LZ and FZ collected and analyzed the references. LL, YY, and YZ revised the manuscript. All authors approved the final manuscripts as submitted.

## Conflict of Interest

The authors declare that the research was conducted in the absence of any commercial or financial relationships that could be construed as a potential conflict of interest.
